# Missed opportunities for integrated testing of HIV and tuberculosis on the GeneXpert platform in Lesotho

**DOI:** 10.4102/ajlm.v12i1.2132

**Published:** 2023-08-28

**Authors:** Gamuchirai P. Gwaza, Monkoe Leqheka, Tsietso Mots’oane, Sabine Dittrich, Kekeletso Kao

**Affiliations:** 1 Department of Impact, Foundation for Innovative New Diagnostics (FIND), Geneva, Switzerland; 2 Department of Continuing Education, University of Oxford, Oxford, United Kingdom; 3 Department of Research and Laboratory Services, Ministry of Health, Maseru, Lesotho; 4 Department of Global Public Health, Deggendorf Institute of Technology, Deggendorf, Germany; 5 Department of Access, Foundation for Innovative New Diagnostics (FIND), Geneva, Switzerland

**Keywords:** Tuberculosis, HIV, TB, GeneXpert, testing, integrated diagnosis, Lesotho

## Abstract

**Background:**

Integrated testing, treatment and care are key strategies for addressing the dual burdens of tuberculosis and HIV. The GeneXpert instrument allows simultaneous HIV and tuberculosis testing, but its utilisation for integrated testing remains suboptimal.

**Objective:**

The study determined the extent to which tuberculosis testing and HIV early infant detection (EID) were integrated on the GeneXpert platform, or the potential for integration at selected health facilities.

**Methods:**

A mixed methods evaluation was conducted using retrospective secondary data analysis of laboratory records from 2017 to 2019, and semi-structured interviews. Data were collected between January 2020 and March 2020 in Lesotho.

**Results:**

Forty-four health staff were interviewed across 13 health facilities: one regional, nine district, and three clinic level. Six were government facilities, six were mission hospitals, and one was a non-profit clinic. All facilities selected had at least one GeneXpert instrument used for tuberculosis or HIV testing; none included simultaneous testing for tuberculosis and HIV. In 2017, the average utilisation rate for the GeneXpert instrument for tuberculosis and EID testing was 63% and 24%, while in 2019, the average utilisation rate was 61% for tuberculosis testing and 27% for EID.

**Conclusion:**

Except for three sites where the testing rates were high, utilisation rates were sufficiently low that all the HIV EID and tuberculosis tests undertaken in 2017 and 2019 could have been performed using only the instruments currently dedicated to tuberculosis testing. There is a missed opportunity for the integration of testing for tuberculosis and HIV on the GeneXpert instrument.

**What this study adds:**

This study adds to the body of evidence on the need for integration of testing and highlights some practical and technical considerations for successful implementation of integrated tuberculosis and HIV testing.

## Introduction

Integrated testing can improve health outcomes, save costs, and improve patient experiences.^1,2,3,4^ Tuberculosis is the leading cause of death among people living with HIV, and people living with HIV are 18 times more likely to fall ill with tuberculosis.^5^ In 2020, 9% of the people diagnosed with tuberculosis lived with HIV.^6^ Implementing integrated testing, treatment, and care activities has been identified as a key strategy for addressing the dual burdens of tuberculosis and HIV.^7,8^ However, integrating testing for HIV and tuberculosis is still suboptimal due to various factors, including limited resources, a lack of clarity on effective integration models, and a lack of coordination among disease programmes.^9^ These factors are common challenges in integrating testing for other disease conditions, such as febrile illnesses.^10^

In 2010, the World Health Organization (WHO) recommended using the Xpert^®^ MTB/RIF assay based on the GeneXpert multidisease platform for diagnosing tuberculosis. The Xpert^®^ MTB/RIF is a simple and robust test making it feasible for use by people with limited or no laboratory training in peripheral laboratories and clinics.^11,12,13^ In 2016, WHO also approved several point-of-care (POC) assays for HIV diagnostics, including the Xpert HIV-1 Qual for HIV qualitative testing for early infant diagnosis (EID) using whole blood or dried blood spots, which used the same GeneXpert platform.^14^ Point-of-care testing provides the opportunity to reduce turnaround times (TAT), limiting patient loss along the HIV testing cascade, reducing infant mortality, and allowing for task shifting to lower cadres of health workers at decentralised facilities.^15,16,17^ The GeneXpert instrument can simultaneously test multiple pathogens, such as tuberculosis and HIV, using different cartridges on the same instrument. The platform has also recently been used for coronavirus disease 2019 testing.^18^ As such, the GeneXpert platform can potentially improve service delivery efficiencies and reduce costs to the patient by integrating testing for tuberculosis and HIV.^19^

Despite significant progress, Lesotho still grapples with the dual burden of tuberculosis and HIV.^18^ Progress on mother-to-child transmission of HIV was bolstered by introducing the POC EID tests,^20^ including the GeneXpert HIV-1 Qual and m-PIMA HIV-1/2 Detect/Alere q.^16^ Lesotho is among the 30 countries with a high tuberculosis burden and has the highest estimated incidence rate of 654 per 100 000 people.^18^

The Foundation for New Innovative Diagnostics has supported the Ministry of Health in Lesotho in rolling out tuberculosis testing on the GeneXpert platform since 2016. Lesotho’s investment in molecular tuberculosis and HIV testing has progressively increased since this time, and the country has a total of 42 GeneXpert instruments with four modules (GXIV) in 21 health facilities that could be used for tuberculosis and HIV testing and 15 Alere Q instruments in 15 health facilities for HIV POC testing.^21^ Due to the high tuberculosis and HIV co-infection rates, Lesotho adopted a strategy of using Xpert^®^ MTB/RIF as the primary diagnostic test for presumptive tuberculosis patients to increase the chances of tuberculosis detection.^21^ The country provided HIV viral load testing for all HIV-positive individuals per the WHO HIV treatment guidelines.^20^ It also offered HIV EID testing at POC to minimise loss of follow-up.

The Lesotho Ministry of Health guidelines for HIV EID testing are consistent with the WHO guidelines, recommending that the first virological test for infants exposed to HIV should be conducted at or around 6 weeks following birth and again after they are weaned from breastfeeding and all infants diagnosed with HIV should be started on anti-retroviral therapy immediately, irrespective of CD4 count.^22^ The WHO also strongly recommended that Xpert^®^ MTB/RIF be used as the initial diagnostic test in individuals suspected of multidrug-resistant tuberculosis or HIV-associated tuberculosis.^23^

The Foundation for New Innovative Diagnostics has been supporting efforts to promote testing integration where possible through conducting various diagnostic network optimisation activities in Lesotho.^24,25^ This evaluation aimed to determine the implementation rate or potential for integrated tuberculosis and HIV testing on the GeneXpert platform in Lesotho from 2017 to 2019. The evaluation focused on HIV EID and tuberculosis testing, as these were the only tests being performed on the GeneXpert in the health facilities at the time of the evaluation in January 2020.

## Methods

### Ethical considerations

No formal ethical approval was required as this was part of a routine programme evaluation. We received permission from the Ministry of Health, Department of Research and Laboratory Services, and a representative from the department was part of the evaluation team. Several ethical measures were taken to protect the data and interviewees, including anonymising their responses and providing aggregate numbers of tests. The interviewees provided informed consent and the interviews were conducted in a confidential space to ensure privacy. No patients were interviewed in this evaluation.

### Study design

Integrated testing was defined in the evaluation to mean that different tests, that is, tuberculosis and HIV, could be conducted at the same time or consecutively on the same GeneXpert instrument. A mixed methods evaluation design was developed, integrating different methods to collect data on defined processes and outcome indicators. Data were collected simultaneously with one researcher conducting interviews while the other reviewed the laboratory records at the health facilities. This data collection was conducted between 13 January 2020 and 24 January 2020. Further data requests for missing or data unavailable in the field were shared between February 2020 and March 2020. The focus was on the Cepheid Xpert^®^ MTB/RIF, referred to as the GeneXpert instrument.

#### Secondary data analysis

Data were collected from laboratory records or files with data for tuberculosis and HIV EID tests conducted at the facility for the most recent period from 2017, 2018 and 2019. Data for 2017 and 2019 were used in the evaluation as they were more complete and could be used for comparisons across the health facilities. Complete data meant that the data included the day the test was taken, time started, time finished, result of the state, and error rates.

#### Semi-structured interviews

These were conducted with the guide of a survey instrument consisting of two questionnaires. The ‘laboratory questionnaire’ covered questions related to GeneXpert instrument installation, staff training, instrument maintenance, quality management, data reporting, testing rates, costs and challenges faced in implementing tuberculosis and HIV testing. The ‘clinical questionnaire’ focused on patients’ general profile, economic costs, staff training, testing rates, linkage to treatment, health outcomes, and challenges related to tuberculosis and HIV patient management. The clinical questionnaire was also meant to provide more details on the health workers’ experience and how they perceived the patients’ experiences.

The inclusion criteria for the interviews were that those interviewed had to be responsible for conducting the tuberculosis or the HIV test on the GeneXpert instrument or were working directly on the tuberculosis or HIV programme on a day-to-day basis. At each site, two people were interviewed working in the lab for tuberculosis or where the GeneXpert for tuberculosis was located and two people working in the maternal health division where the GeneXpert was located for HIV EID testing.

The questionnaires were developed based on the metrics that the Foundation for New Innovative Diagnostics had been using to monitor the GeneXpert implementation within the facilities prior to 2016 where they had provided funding for the activities. As such, they had been piloted and tested within the same contexts.

### Setting

Lesotho employs a hub and spoke model for sample referrals. The hubs are laboratories often located in hospitals, either at the district or regional level, where the GeneXpert instrument is located.^21^ Samples are referred from collection sites’ ‘spokes’ to testing sites’ ‘hubs’. The spokes are clinics or health centres where samples are collected and transported to the hubs for testing.^26^ Generally, the hubs in peri-urban areas cover a large catchment area and receive samples from multiple spokes.

### Sampling strategy

GeneXpert testing sites in rural, urban, and peri-urban areas across the country were selected based on discussions with the Ministry of Health staff to ensure diversity in region and type of facility. The health facilities were purposely sampled to represent the geographic breadth and size of the facilities, that is, peri-urban, rural and urban sites and regional, district, and local hospitals and clinics.^3^ The facilities selected had to have at least one GeneXpert instrument used for tuberculosis or HIV testing. In some cases, the facility used GeneXpert for tuberculosis testing and the Alere Q for EID testing. These were still included in the evaluation and the data were later excluded from the analysis to allow for better comparison.

### Data analysis

The data were analysed in Microsoft Excel (Microsoft Corp., Redmond, Washington, United States). The utilisation rate for the GeneXpert instrument was calculated by dividing the actual number of tests performed each year (for a given site and disease) by the maximum possible number of tests that could be performed. The WHO guidelines recommend that the maximum capacity of a single, four-module GeneXpert instrument is 20 specimens per day.^23^ However, the recent Global Laboratory Initiative guide recommends that one technician could perform more than 12 Xpert^®^ MTB/RIF tests per day.^27^ For practical consideration, a maximum capacity of 12 specimens per day was used in the analyses, for a single GXIV, given three 2-h runs in a day. An assumption of 264 days of testing per annum, based on 22 days per month recorded during data collection at the sites, was used. Based on these calculations, the maximum capacity of a single, four-module GeneXpert instrument is 3168 specimens each year (12 specimens/day × 264 days).

We defined the standard TAT in two distinct ways: (1) the time from sample collection to results being received in the laboratory and (2) the time from sample collection to when the patient receives results. These vary by countries and sites and are usually determined for each based on infrastructure capacity. The TAT was collected through interviews. This is because laboratory records showed the time when the sample was received and when the test was completed and did not always show when the patient collected the results. During the interviews the staff were asked, ‘Approximately how long does it take from the time the sample is collected to when the patient receives their results?’ If they could, they were asked to break down the times into the two stages defined above.

## Results

### Sample

In total, 44 people were interviewed (24 tuberculosis interviews, 18 HIV interviews, and two Ministry of Health interviews), with an average of two people per site and two in the central Ministry of Health offices. We completed 42 questionnaires; 24 for tuberculosis and 18 for HIV. Of the tuberculosis and HIV questionnaires, half focused on the clinical aspects, while the other half focused on the laboratory questionnaire.

Thirteen health facilities were included in the evaluation (Table 1). Twelve health facilities conducted tuberculosis testing on the GeneXpert instrument, (one site only had no tuberculosis testing: Thamae Health Centre) and they served a combined total of 137 spoke sites. Nearly half (46%; 6/13) of the hospitals had more than one GXIV, such as Mafeteng (*n* = 3), Berea (*n* = 2), Motebang (*n* = 2), Ntshekhe (*n* = 2), Partners in Health Clinic (*n* = 2) and Scott Hospital (*n* = 2). Nine health facilities had a GXIV for HIV EID testing and 73 spoke sites.

**TABLE 1:** Location of health facilities visited in Lesotho in January 2020.

Health facility	Location		No. of four-module GeneXpert instruments		No. of spoke sites	
	District	Setting	Tuberculosis	HIV EID	Tuberculosis	HIV EID
Berea Hospital	Berea	Peri-urban	2	1	12	12
Maluti Hospital	Berea	Rural	1	1	7	7
Mafeteng Hospital	Mafeteng	Peri-urban	3	1	9	12
Maputsoe Filter Clinic	Leribe	Peri-urban	1	n/a	0	n/a
Motebang Regional Hospital	Leribe	Peri-urban	2	1	31	9
Seboche Hospital	Leribe	Rural	1	1	5	5
Ntshekhe Hospital	Mohale’s Hoek	Peri-urban	2	1	25	8
Paray Hospital	Thaba Tseka	Rural	1	1	11	8
Partners in Health (PIH) Laboratory	Maseru	Urban	2	n/a	7	n/a
Scott Hospital	Maseru	Peri-urban	2	1	15	10
Thamae Health Centre	Maseru	Urban	-	1	-	2
St James Hospital	Mokhotlong	Rural	1	n/a	7	n/a
St Joseph’s Hospital	Roma	Peri-Urban	1	n/a	8	n/a

**Total**	**-**	**-**	**19**	**9**	**137**	**73**

Note: Only GeneXpert instruments are listed. Alere Q instrument placement has been left out of this report and marked as n/a in the table.

EID, early infant detection; n/a, not applicable; No., number.

None of the 13 sites had integrated tuberculosis and HIV EID testing on the same instrument, Instead, tuberculosis and HIV testing were conducted separately in different rooms by different people on different instruments.

In 2017, there were 32 037 tuberculosis tests conducted on 16 GXIV machines (Table 2). Seven GeneXpert instruments for EID testing were installed in 2017. There were data available from three of the sites; a total of 2297 EID tests were conducted. Overall utilisation rates for the three sites ranged from 54% to 72%. In 2017, the average utilisation rate for the GeneXpert instrument for tuberculosis and EID testing was 63% and 24%.

**TABLE 2:** Number of tests conducted on the GeneXpert instrument, based on the data recorded in lab registers and hospital files.

Health facility	2017					2019				
	Tuberculosis	EID	Total	Maximum annual capacity†	Utilisation rate (%)	Tuberculosis	EID	Total	Maximum annual capacity	Utilisation rate (%)
Berea Hospital	2565	n/a	-	-	-	2553	846	3399	6336	54
Maluti Hospital	1849	442	2291	3168	72	2948	690	3638	3168	115
Mafeteng Hospital	4347	1321	5668	9504	60	4565	1033	5598	9504	59
Maputsoe Filter Clinic	n/a	n/a	-	-	-	44§	381	-	-	-
Motebang Regional Hospital	5959	n/a	-	-	-	8573	835	9408	6336	148
Seboche Hospital	1054	n/a	-	-	-	1476	340	1816	3168	57
Nshekhe Hospital	3038	n/a	-	-	-	3834	834	4668	6336	74
Paray Hospital	1165	534	1699	3168	54	1699	549	2248	3168	71
Partners in Health (PIH) laboratory	6061	n/a	-	-	-	4883	n/a	-	-	-
Scott Hospital	3710	n/a	-	-	-	3974	703	4677	3168	148
Thamae Health Centre	n/a	n/a	-	-	-	766	766	-	-	-
St James Hospital	664	n/a	-	-	-	644	‡	-	-	-
St Joseph’s Hospital	1625	n/a	-	-	-	1432	‡	-	-	-

**Total**	**32 037**	**2297**	**-**	**-**	**-**	**37 347**	**6977**	**-**	**-**	**-**

Note: Calculation of utilisation rate: (Total of tuberculosis + EID tests conducted) / Maximum capacity × 100%. For facilities with more than one GeneXpert instrument, the number of tests performed per year was divided by the number of instruments to get an estimate of the utilisation rate of one instrument. The percentage is rounded off to the nearest 100. n/a, data not available either because the GeneXpert was not available to do the HIV EID tests or in some cases tuberculosis or HIV EID tests were not conducted at the facility.

EID, early infant detection; n/a, not applicable.

† , Maximum capacity for the GXIV was calculated based on the assumption that 12 specimens were run per day, given three 2-h runs in a day. In addition, 264 days of testing per annum was used, based on 22 days per month recorded during data collection at the sites, was used, that is, 264 × 12 = 3168 tests per year for one instrument. This was multiplied by 2, 3 or 4 depending on the number of GXIV modules available at the facility;

‡ , Results excluded because tests were conducted on the Alere Q instrument not the GeneXpert instrument;

§ , December-only data, excluded from the addition as it is not complete.

In 2019, there were 19 GXIV instruments used for tuberculosis testing across 12 sites, with a total of 37 347 tuberculosis tests conducted. Nine GXIV instruments were used for EID testing across nine sites, and 6977 EID tests were conducted. Overall utilisation rates ranged from 54% to 148%. In 2019, the average utilisation rate was 61% for tuberculosis testing and 27% for EID.

#### Turn-around time

The TAT for performing the tests and receiving the results in the laboratory is 2 h on the GeneXpert instrument. The time to get results to the patients at the hub site ranged from 2 h to 24 h across the sites for both tuberculosis and HIV testing (Table 3). The average time to get the results to the patients in the spoke sites varied across the sites and ranged from 2 to 7 days.

**TABLE 3:** Turn-around time for return of tuberculosis and early infant detection test results to patients at hub and spoke sites, Lesotho, January 2020 – March 2020.†

Name of health facility	Hub sites (hours)		Satellite sites (hours)	
	Tuberculosis	EID	Tuberculosis	EID
Berea Hospital	3	2	48	48
Maluti Hospital	2	2	168	168
Mafeteng Hospital	2	2	48	48
Maputsoe Filter Clinic	2	‡	n/a	n/a
Motebang Regional Hospital	2	2	48	168
Seboche Hospital	2.5	2	120	120
Nshekhe Hospital	3	1.5	48	48
Paray Hospital	2	2	48	48
Partners in Health (PIH) laboratory	2	n/a	120	n/a
Scott Hospital	24	24	168	168
Thamae Health Centre	n/a	2	n/a	48
St James Hospital	2	2	48	48
St Joseph’s Hospital	24	‡	48	‡

Note: n/a shows tests not conducted at the facility.

EID, early infant detection; n/a, not applicable.

† , Results based on data collected from interviews with health workers because laboratory records on patient receipt of results were incomplete;

‡ , Results excluded because tests were conducted on the Alere Q instrument not the GeneXpert instrument.

#### Staff capacity

The number of staff trained to operate the GeneXpert instrument varied across sites and diseases (Table 4). On average, there were four trained staff for tuberculosis testing and three for HIV testing on the GeneXpert instrument. For the tuberculosis testing, staff trained were usually laboratory technicians, microscopists, or health technologists. Tuberculosis testing was conducted within the central laboratory at the health facility. On the other hand, for HIV EID testing, nurses, professionals, and lay counsellors were trained to operate the GeneXpert instrument. The EID testing was done at the POC within the maternal wing of the health facility.

**TABLE 4:** Staff trained to operate the GeneXpert instrument for testing, Lesotho, January 2020 – March 2020.†

Name of health facility	No. of trained staff	
	Tuberculosis	EID
Berea Hospital	7	2
Maluti Hospital	2	3
Mafeteng Hospital	4	6
Maputsoe Filter Clinic	1	‡
Motebang Regional Hospital	4	3
Seboche Hospital	3	3
Nshekhe Hospital	3	4
Paray Hospital	3	2
Partners in Health (PIH) laboratory	6	n/a
Scott Hospital	6	2
Thamae Health Centre	n/a	4
St James Hospital	4	‡
St Joseph’s Hospital	6	‡

Note: n/a shows tests not conducted at the facility.

EID, early infant detection; n/a, not applicable; No., number.

† , Results based on data collected from interviews with health workers;

‡ , Results excluded because tests were conducted on the Alere Q instrument not the GeneXpert instrument.

### Linkage to treatment

In all of the HIV EID sites, there was same-day initiation of treatment, with 100% of those testing positive being initiated on anti-retroviral therapy. The proportion of infants staying on treatment for at least 12 months was very high (100%) across six sites. For HIV satellite sites, five of the sites had same-day initiation of treatment (Seboches, Ntsheke, Scott, Motebang, Thamae), while two (Paray and Mafeteng) had 2 days, one site (Berea) had 4 days, and one site (Maluti Hospital) could be up to 10 days (Table 5). Care cascade and linkage to treatment data for tuberculosis were not complete or available for more than half of the facilities (7 out of 12) and were therefore excluded from the results.

**TABLE 5:** Time for patient linkage to treatment for HIV early infant detection, Lesotho, January 2020 – March 2020†.

Name of health facility	Average time between testing and initiating anti-retroviral therapy (ART) in days (on site)		Percentage of those on ART who remain on ART for at least 12 months (%)
	Hub	Satellite	
Berea Hospital	Same day	4 days	100
Maluti Hospital	Same day	10 days	100
Mafeteng Hospital	Same day	2 days	100
Maputsoe Filter Clinic	‡	n/a	n/a
Motebang Regional Hospital	Same day	Same day	100
Seboche Hospital	Same day	Same day	100
Ntshekhe Hospital	Same day	Same day	n/a
Paray Hospital	Same day	2 days	n/a
Partners in Health (PIH) laboratory	n/a	n/a	n/a
Scott Hospital	Same day	Same day	80
Thamae Health Centre	Same day	Same day	100
St James Hospital	‡	n/a	n/a
St Joseph’s Hospital	‡	n/a	n/a

Note: n/a means no data available.

n/a, not applicable.

† , Results based on data collected from interviews with health workers;

‡ , Results excluded because tests were conducted on the Alere Q instrument not the GeneXpert instrument.

### Barriers to integrating tuberculosis and HIV testing on the same platform

#### Resources

**Donor alignment:** One barrier to integration noted during the interviews was the lack of coordination among the different international funding partners for tuberculosis and HIV. In many cases, each funder purchased their instrument and had different reporting requirements, making it challenging to integrate the workflow and testing even if the devices were housed in adjacent rooms and could have been used by both programmes.

**Time:** Another barrier to integration was potential time constraints if testing was increased, especially in sites with a higher utilisation rate.

#### Training

Another concern raised in the interviews was that staff conducting HIV training had little training in tuberculosis testing and vice versa. Some had received the training in the past but had yet to have a chance to put it into practice and would need more training before they could conduct the tests.

#### Information technology connectivity

Another issue mentioned was the need for a functional data connectivity system to record information and transmit results electronically to clinicians, which would have made integration easier. Some sites had limited data availability as they still relied on a paper-based system or were transitioning to a digital system. Most of the GeneXpert instruments were no longer linked or could use e-reporting. As a result, staff entered results manually, placing an extra burden on them.

## Discussion

In this evaluation of integrated testing of tuberculosis and HIV on the GeneXpert platform, none of the sites visited tested for tuberculosis and HIV on the same instrument, and most instruments were not operating at maximum capacity. This has been observed not just in Africa but also in Europe, because although the WHO European region has moved towards integration, this has had a limited effect on the programmatic organisation for tuberculosis and HIV nationally, which is partly dependent on donor funding that is often project-related and focused on a particular disease.^13^

The utilisation rates in our study were consistent with results from a previous diagnostic network mapping exercise in 2017 across the 10 districts of Lesotho, which had shown low utilisation of GeneXpert instruments, with 19 out of 24 facilities sampled using instruments at less than 50% capacity.^24^ This underutilisation of GeneXpert instruments for tuberculosis has been reported in a trend analysis of 22 tuberculosis high-burden countries with just seven countries (37%) (South Africa, Tanzania, Ethiopia, Nigeria, Pakistan, Uganda, and Zimbabwe) utilising the instruments for multiple tests, including tuberculosis and other diseases, such as HIV and hepatitis-C virus.^28^ Similarly, for HIV EID, underutilisation was observed in another study in Zimbabwe in 2019, where the average daily utilisation of POC EID instruments was 1.51 tests/day.^29^ A later study (2020) in Lesotho confirmed that the GeneXpert instruments are largely underutilised, and relocation of instruments will deliver equivalent access to services compared to procuring new instruments.^25^

The relatively low utilisation rate at many of the sites evaluated in this study suggests there is potential for integrated testing in future, purely based on capacity. For example, in 2017, the three sites that installed new GeneXpert instruments could have integrated the EID tests on the available GeneXpert machines. At Mafeteng, with the two machines available for tuberculosis, there would have been a total of 5668 tests (4347 for tuberculosis and 1321 for EID) out of a possible maximum of 6336 tests, which would have been an 89% utilisation rate. Similarly, in Maluti, 2291 tests (1849 tuberculosis and 442 EID) could have been conducted on one instrument with a 72% utilisation rate. In Paray, 1699 (1165 tuberculosis; 534 EID) tests could have been undertaken at a 53.6% utilisation rate. In 2019, had all the EID tests been integrated or conducted on the GeneXpert instruments used for tuberculosis testing, five sites would still not have used the maximum capacity. Only three of the sites would not have been able to handle both tests without an additional machine (Table 2).

Integrating testing across major diseases can help leverage resources in developing countries.^30^ The feasibility of integrated testing was successfully demonstrated in 2018 in a study in Zimbabwe on the GeneXpert platform for tuberculosis testing, detecting resistance to the antibiotic rifampicin, EID, and HIV viral load testing in the treatment monitoring of patients on anti-retroviral therapy.^31^ Fortunately, this integration is already part of Lesotho’s US President’s Emergency Plan for AIDS Relief (PEPFAR) country support plan within the tiered laboratory network.^20^ However, there are still barriers to operationalising integrated testing in Lesotho, which are common in other countries. For example, staff capacity was also identified as a barrier to tuberculosis and HIV integration in a 2009 paper in Uganda. Health workers had been trained on tuberculosis or HIV and not on tuberculosis-HIV integration. In addition, follow-up supervision and monitoring after the training were minimal.^9^

The GeneXpert instrument and many POC tests can improve patient experiences by reducing waiting times. In this evaluation, all the hub sites had a TAT, that is, patients getting their results, of less than 4 h, consistent with similar studies that had shown same-day results return to a caregiver at testing sites and a TAT of 5 days at spoke sites, a significant improvement over the conventional model of 61.7 days.^26^ The TAT noted in this evaluation was a reduction from a 2019 in a study in eight African countries that had seen a TAT of 9 days at spoke sites.^16^ This reduction could have responded to a country review of the joint tuberculosis and HIV programme, which noted that the TAT in some spoke sites needed urgent attention as it could take up to 2 weeks.^32^ Reducing TAT can increase the testing rates for tuberculosis and HIV as it will cut down on travel time and costs, especially in rural areas, which may be a significant burden for some people.

However, informal discussions with some laboratory workers during the evaluation showed that even though technically the TAT for getting results to the patients at the hubs could be between 2 h and 3 h, which is the time it took to run the test, in practice the patient still had to wait at least a day to get their results. This was because the lab workers did not want the added pressure of patients waiting for their results as they had a lot of tests from their spoke sites to attend to. The time commitment by the patients constitutes an additional economic cost, precluding the patients and those accompanying them from doing other paid work or caring duties for several hours or days. Interventions are needed to increase service coverage and utilisation of services in these areas.

### Policy implications

This evaluation highlights areas that need more focus and more detailed diagnostic network optimisation exercises to improve service delivery and allocate stocks, staff, equipment, and training. Better coordination of donor support or increased flexibility in reporting of use will be essential to maximise the use of resources, where integration may be more effective than purchasing new equipment. Monitoring and evaluation should be a routine part of the implementation and should be budgeted and demanded by policymakers and donors. Health facilities may require additional resources and technical support to improve data collection, analysis, and dissemination.

### Limitations

Access to data was a challenge in some sites, which affected the analysis. The evaluation focused on 2017 and 2019 data only, which was the most complete. Due to a lack of variation in the cost data by site, a cost-effectiveness analysis could not be done.

### Conclusion

Previous reviews and studies have shown that integrated testing is feasible and can improve health outcomes and patient experiences. The findings from this evaluation show that there are still operational challenges to implementing integrated testing. An understanding and further research of how and why integrated testing models work and in which contexts will allow for effective targeted interventions to improve testing coverage across several diseases especially taking advantage of the multidisease testing platforms.
